# A Non‐Covalent [4Fe–4S]/[2Fe] Interface in HydF Guides [FeFe]‐Hydrogenase Maturation

**DOI:** 10.1002/anie.8078898

**Published:** 2026-05-29

**Authors:** Giorgio Caserta, Princess R. Cabotaje, Armel T. Waffo, Deepak Prajapat, Ilya Sergueev, Stefan Frielingsdorf, Gustav Berggren

**Affiliations:** ^1^ Institut für Chemie Technische Universität Berlin Berlin Germany; ^2^ Department of Chemistry─Ångström Laboratory Molecular Biomimetics Uppsala University Uppsala Sweden; ^3^ Okinawa Institute of Science and Technology‐Evolution Cell Biology, and Symbiosis Unit Okinawa Japan; ^4^ Department of Cell and Molecular Biology – Molecular Evolution Uppsala University Uppsala Sweden; ^5^ Deutsches Elektronen‐Synchrotron Hamburg Germany

**Keywords:** biosynthesis, hydrogenase, iron‐sulfur clusters, metalloenzymes, nrvs

## Abstract

Maturation of [FeFe]‐hydrogenase depends on the HydF maturase, a [4Fe–4S]‐containing scaffold protein that assembles and delivers the organometallic [2Fe] subsite of the H‐cluster. Despite extensive research, the function of the HydF [4Fe–4S] cluster and its interaction with the [2Fe] cofactor remains unresolved, with conflicting evidence regarding cyanide linkage isomerism and the functional role of the cubane. Using ^57^Fe nuclear resonance vibrational spectroscopy in combination with selective isotopic labeling, we show that the [2Fe] subsite binds adjacent to the [4Fe–4S] cluster without forming a covalent cyanide bridge, while remaining electronically coupled. Complementary protein structure predictions support this arrangement and reconcile prior spectroscopic, mutagenesis, and structural studies. Extending this framework to earlier biosynthetic steps, additional structure predictions suggest that the [4Fe–4S] cluster contributes to assembly of the CH_2_–NH–CH_2_ bridge of the [2Fe] site via interactions with the lipoate cofactor of the aminomethyl‐lipoyl H‐protein, thereby positioning reactive components in proximity to the [2Fe] precursor. Together, these results provide a coherent structural and mechanistic framework for HydF function across distinct stages of H‐cluster biosynthesis.

## Graphical abstract

Bridged or not? ^57^Fe NRVS reveals a non‐covalent, electronically coupled interaction between the HydF [4Fe–4S] cluster and the [2Fe] precursor. Boltz‐2 structure predictions suggest that the cubane organizes lipoate‐dependent H_met_ chemistry, positioning intermediates for assembly of the CH_2_–NH–CH_2_ bridge during H‐cluster biosynthesis.

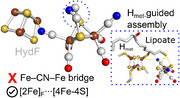

## 1 Introduction

**FIGURE 1:**
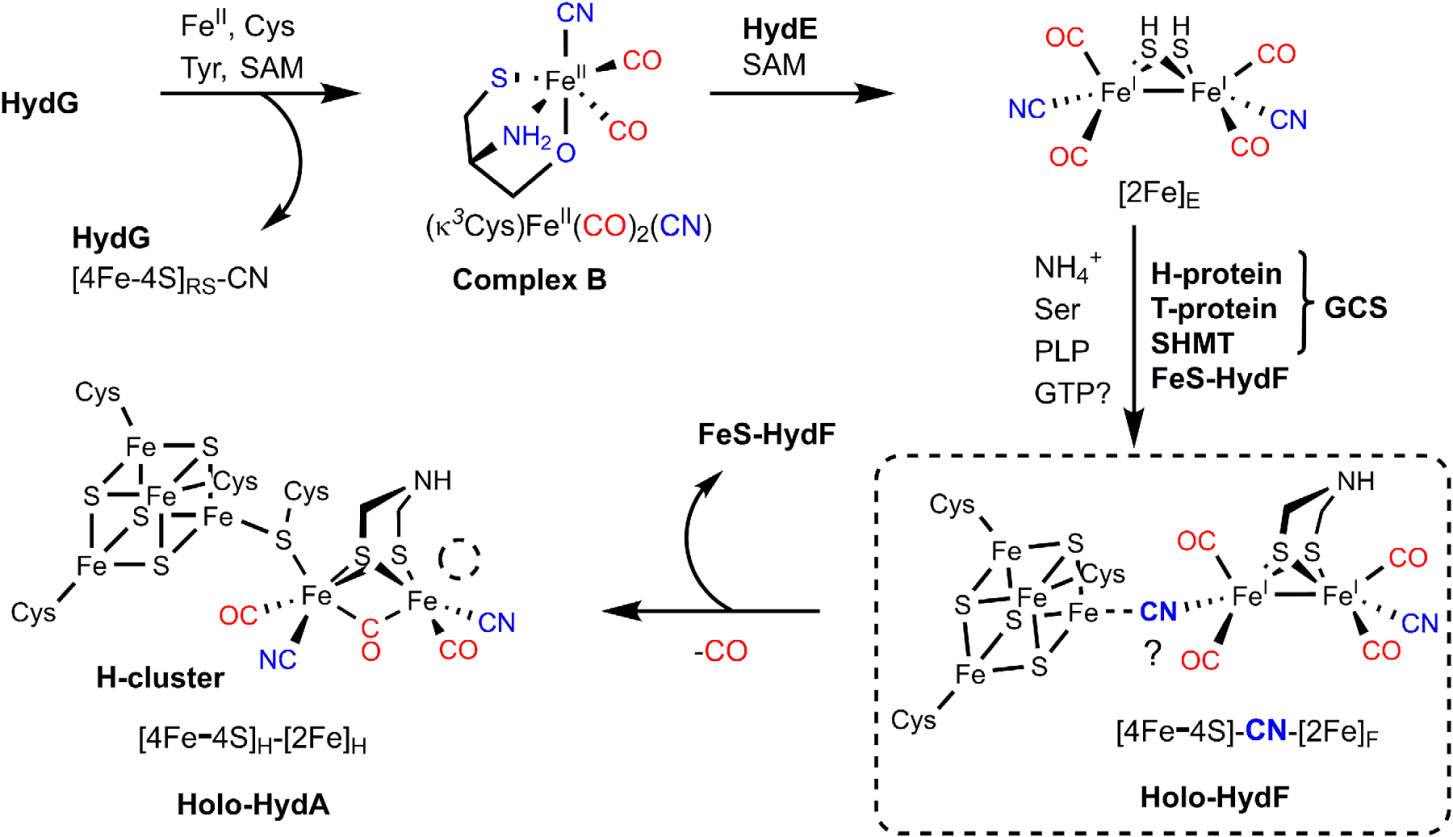
Schematic overview of the [FeFe]‐hydrogenase maturation pathway and proposed interaction between the HydF cofactors. Maturation is initiated by the rSAM enzyme HydG, which converts tyrosine into CO and CN^–^ ligands and generates an Fe^II^(κ^3^‐Cys)(CO)_2_(CN) synthon (complex B). Complex B is then transferred to the rSAM enzyme HydE, where reductive activation yields a low‐spin Fe^I^ intermediate that dimerizes to form [2Fe]_E_. In the presence of HydF and the aminomethyl‐lipoyl H‐protein (H_met_), [2Fe]_E_ is further converted into the fully functional [2Fe]_F_ precursor via installation of the DTMA bridge. Earlier ^15^N HYSCORE and ^13^C ESEEM studies on [2Fe(pdt)]_F_(^13^CN)‐HydF suggested a close interaction between the ^13^C nucleus of a labeled cyanide ligand and the unpaired electron of the [4Fe–4S] cluster, originally interpreted as a cyanide‐mediated contact potentially involving linkage isomerism [20].

[FeFe]‐hydrogenases catalyze the reversible interconversion of protons and dihydrogen using a sophisticated organometallic active site known as the H‐cluster [1, 2]. This cofactor comprises a canonical [4Fe–4S] cubane coordinated by four conserved cysteine residues and a diiron catalytic subsite ([2Fe]_H_) [3]. The [2Fe]_H_ subsite is covalently linked to the [4Fe–4S] cluster via a cysteine thiolate (Figure 1) and is coordinated by three carbon monoxide (CO) ligands, two cyanide (CN^–^) ligands, and an azadithiolate (ADT, also referred to as dithiomethylamine, DTMA) bridge [4]. Whereas the [4Fe–4S]_H_ cluster and additional accessory [Fe–S] centers (F‐clusters) are assembled by the housekeeping iron–sulfur cluster machinery [5], biosynthesis of the [2Fe]_H_ subsite requires a dedicated maturation pathway involving the HydE, HydF, and HydG proteins (Figure 1) [6, 7]. Maturation is initiated by the radical S‐adenosyl‐L‐methionine (rSAM) enzyme HydG, which catalyzes the conversion of tyrosine into CO and CN^–^ ligands and generates an Fe^II^(κ^3^‐Cys)(CO)_2_(CN) synthon [8, 9, 10]. This iron precursor, commonly referred to as complex B, is transferred from HydG to a second rSAM enzyme [11, 12], HydE, which mediates reductive activation of the Fe^II^ synthon to form an adenosylated low‐spin Fe^I^ intermediate [13, 14]. This species subsequently dimerizes to yield the [Fe^I^Fe^I^] complex [Fe_2_(HS)_2_(CO)_4_(CN)_2_]^2–^, denoted [2Fe]_E_ [15]. Rauchfuss, Britt, and coworkers further showed that synthetic [2Fe]_E_ efficiently matures apo‐HydA1 from *Chlamydomonas reinhardtii* in the presence of the third maturase HydF and *Escherichia coli* cell lysate [16]. Building on this work, Broderick and coworkers identified a fourth essential component of the maturation pathway: the aminomethyl‐lipoyl H‐protein (H_met_) of the glycine cleavage system. H_met_ catalyzes installation of the CH_2_–NH–CH_2_ moiety across the two bridging sulfides of [2Fe]_E_, generating the fully functional diiron precursor [Fe_2_(CO)_4_(CN)_2_(adt)]^2–^, termed [2Fe]_F_ [17, 18, 19].

Importantly, installation of the azadithiolate bridge occurs exclusively when the [2Fe]_E_ precursor is bound to HydF, underscoring the central role of this protein in [FeFe]‐hydrogenase maturation [21]. HydF is a guanosine triphosphatase (GTPase) that harbors a [4Fe–4S] cluster, which has been extensively characterized [22, 23, 24, 25]. An X‐ray crystal structure of HydF from *Thermosipho melanesiensis* (*Tme*HydF) revealed that this cluster is coordinated by three cysteine residues and a glutamate [26]. Despite these structural insights, the functional role of the [4Fe–4S] cluster remains the subject of ongoing debate [17, 27]. In 2013, we showed that synthetic [2Fe]_F_ analogues bearing dithiolate bridges with different bridgehead groups (CH_2_, NH, or O) can be loaded onto HydF from *Thermotoga maritima* (*Tm*HydF) and subsequently support maturation of apo‐*Cr*HydA1 [20]. Advanced EPR studies suggested a close interaction between the synthetic diiron cofactor and the HydF [4Fe–4S] cluster, which was interpreted as involving a cyanide‐mediated contact, potentially consistent with linkage isomerism (Figure 1) [20, 26]. In this context, the Darensbourg group demonstrated that cyanide flipping can occur in cyanide‐bridged diiron model systems such as Fe–CN/NC–Cu^I/II^ complexes [28, 29]. Later, Happe and coworkers reported that the cubane is dispensable for the final stages of the maturation, as apo‐HydF loaded with synthetic [2Fe]_F_ was still able to activate apo‐*Cr*HydA1 [30]. Conversely, Broderick and coworkers concluded that the [4Fe–4S] cluster is essential, demonstrating that only cluster‐containing HydF supports the final stages of [2Fe]_F_ biosynthesis [31]. These latter findings are consistent with earlier work by King and coworkers [32], which likewise established the importance of the cluster for in vivo H‐cluster assembly. Thus, functional studies have yielded conflicting conclusions regarding the importance of the HydF [4Fe–4S] cluster.

Structurally, a binding site for the [2Fe]_F_ precursor was first identified by the Fontecave group [26, 33]. The picture was later refined by Happe and coworkers [34], which pinpointed residues critical for binding and assembly of [2Fe]_F_. However, neither these static structural models nor prior spectroscopic data could directly address whether the interaction between the [4Fe–4S] cluster and the [2Fe]_F_ subsite involves a direct covalent (cyanide‐bridged) linkage or a non‐covalent association. This distinction is critical, as it defines fundamentally different functional roles for the cluster, ranging from direct coordination of the [2Fe] subsite to a purely structural and electronic contribution, and is therefore essential for defining the function of HydF within the broader framework of H‐cluster maturation. To directly probe the interaction between the [4Fe–4S] cluster and the [2Fe]_F_ subsite of HydF, we employed nuclear resonance vibrational spectroscopy (NRVS) in combination with structure predictions and protein–ligand docking calculations. NRVS is a synchrotron‐based technique that selectively probes vibrational modes of Mössbauer‐active nuclei (e.g., ^57^Fe) and is exquisitely sensitive to changes in bonding, geometry, and oxidation state of iron atoms [35, 36]. The method has previously provided detailed structural insights into a wide range of iron‐containing enzymes, including hydrogenases [37, 38, 39], nitrogenases [40], heme, [41] and nonheme systems [42, 43]. As such, NRVS provides a unique experimental handle to directly assess whether—and how—the [4Fe–4S] cluster of HydF interacts with the [2Fe]_F_ subsite, beyond the reach of static structural models and conventional ensemble spectroscopies.

## 2 Results and Discussion

**FIGURE 2:**
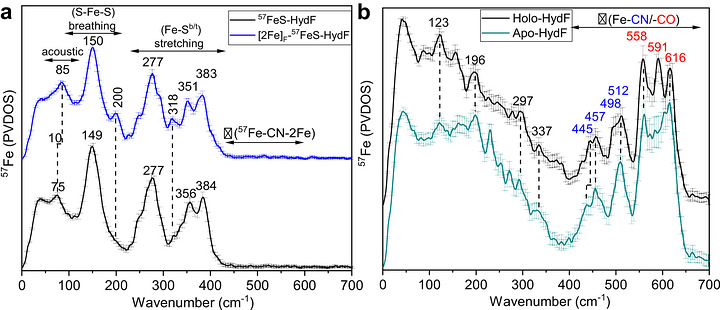
NRVS characterization of selectively labeled [4Fe–4S] and [2Fe]_F_ sites in HydF. (a) NRVS spectra of ^57^FeS‐*Tm*HydF (black trace) and [2Fe]_F_–^57^FeS‐*Tm*HydF (blue trace) exclude the presence of a covalent cyanide bridge or cyanide linkage isomerization between the two iron sites. (b) NRVS spectra of [2^57^Fe]_F_‐FeS‐*Tm*HydF (holo‐HydF, black trace) and [2^57^Fe]_F_‐*Tm*HydF (apo‐HydF, cyan trace) reveal a similar binding mode of [2Fe]_F_ in holo‐ and apo‐HydF.

Here, we report NRVS spectra of holo‐*Tm*HydF in which either the [4Fe–4S] cluster or the diiron subsite was selectively labeled with ^57^Fe. Apo‐*Tm*HydF was isolated as described previously [44], and its [Fe–S] cluster was semi‐synthetically reconstituted under strictly anaerobic conditions (see methods in Supporting Information). UV–vis spectroscopy (Figure S1) and iron quantification (4.7 ±  0.2 Fe/protein) following size‐exclusion chromatography confirmed the assembly of a [4Fe–4S] cluster in *Tm*HydF, hereafter termed FeS‐HydF. To distinguish isotopically enriched samples from those prepared with iron of natural abundance, the former are denoted ^57^FeS−HydF, whereas the latter are referred to as FeS‐HydF. ^57^FeS‐HydF was analyzed as prepared, treated with an excess of sodium dithionite to probe redox‐dependent spectral changes, and a third aliquot was incubated anaerobically with [2Fe]_F_ to yield [2Fe]_F_–^57^FeS‐HydF, denoted holo‐HydF. Unbound [2Fe]_F_ was removed prior to spectroscopic analyses. Infrared spectroscopy of holo‐HydF (Figure S2) displayed the characteristic CO/CN^–^ stretching vibrations associated with protein‐bound [2Fe]_F_, while UV–visible spectra revealed the appearance of a new absorption band at 350 nm, diagnostic of the diiron subsite within the HydF scaffold (Figure S3). Figure 2a compares the NRVS spectra of as‐prepared ^57^FeS‐HydF and holo‐HydF. Both spectra exhibit vibrational features characteristic of a [4Fe–4S] cluster in the 2+ oxidation state, including intense Fe–S stretching modes of the bridging (S^b^) sulfides and terminal thiolate ligands (S^t^) in the ∼250–400 cm^−1^ region [35]. Upon reduction these modes shift by approximately 10–30 cm^−1^ to lower energies (Figure S4), consistent with weakening of Fe–S bonds, whereas lower‐energy S–Fe–S bending and twisting (100–200 cm^−1^), and acoustic modes (<100 cm^−1^) remain largely invariant with respect to the cluster oxidation state.

Notably, the NRVS spectrum of holo‐HydF does not exhibit absorptions in the frequency range expected for Fe_FeS_–CN_2Fe_ stretching modes, which would be diagnostic of cyanide linkage isomerization involving direct coordination to the [4Fe–4S] cluster. The close similarity between the holo‐HydF and ^57^FeS‐HydF spectra therefore argues against the presence of a covalent cyanide bridge between the [2Fe]_F_ subsite and the cubane (Figure 1). Instead, comparison of the spectra reveals additional low‐frequency features in holo‐HydF at 85 and 200 cm^−1^, which fall within the region associated with torsional and breathing modes of the [4Fe–4S] cluster, as well as collective motions of the cubane coupled to the protein backbone, consistent with weak, non‐covalent coupling between the two metal sites. In addition, Fe–S stretching vibrations in ^57^FeS‐HydF are slightly red‐shifted upon [2Fe]_F_ loading (356/351, 384/383 cm^−1^), consistent with weakened Fe─S bonds and increased electron density at the cubane. Holo‐HydF displays also an enhanced absorption at ∼318 cm^−1^ within the Fe─S stretching region. This increased intensity is consistent with a redistribution of vibrational modes within the Fe─S stretching manifold upon [2Fe]_F_ binding. Proximity of the diiron subsite likely perturbs the local electrostatic and structural environment of the cubane, altering normal‐mode mixing and enhancing the ^57^Fe contribution to specific Fe─S stretches without major frequency shifts. Consistent with this interpretation, [2Fe]_F_ binding does not substantially perturb the EPR signature of the [4Fe–4S]^+^ cluster [21, 26], yet reduction of holo‐HydF induces a slight blueshift of the dominant CO stretching band of [2Fe]_F_ [20, 26], indicative of electronic communication between the two metal sites in the absence of covalent linkage.

FeS−HydF was then reacted with [2^57^Fe]_F_ to monitor the complete set of Fe–ligand vibrations of the di‐iron core when bound to the protein scaffold. As a control, we also reacted apo‐HydF with [2^57^Fe]_F_. Iron quantitation, 1.6 ± 0.1 Fe/protein, and UV–visible spectroscopy (Figure S5) confirmed incorporation of [2^57^Fe]_F_. NRVS spectra for these two HydF samples are shown in Figure 2b. Holo‐HydF exhibits intense bands at 558, 591, and 616 cm^−1^, primarily associated with Fe─CO vibrational modes, accompanied by weaker absorptions at ca. 450 and 500 cm^−1^ attributable mainly to Fe–CN vibrations [45]. Owing to selective ^57^Fe labeling of the [2Fe] core, additional low‐frequency features at 123, 196, 297, and 337 cm^−1^ could be resolved that would otherwise be masked by contributions from the [4Fe–4S] cluster.

Importantly, the NRVS data of apo‐HydF closely resemble those of holo‐HydF, demonstrating that [2Fe]_F_ can be successfully loaded into apo‐HydF in a binding mode analogous to that in holo‐HydF [30, 34]. Evidently, this mode of incorporation is consistent with the absence of cyanide linkage isomerism, as apo‐HydF lacks a [4Fe–4S] cluster. Fe_FeS_–NC_2Fe_ coordination in holo‐HydF appears also unlikely [46], as such a linkage would be expected to induce substantial perturbations in both the [4Fe–4S] and [2Fe]_F_ vibrational regions as well as measurable differences between apo‐ and holo‐HydF spectra (Figure 2), neither of which is observed.

However, holo‐HydF spectra display sharper and better‐resolved vibrational features, particularly in the Fe–CO stretching region, which may be indicative of a more homogeneous [2Fe]_F_ binding. Taken together, these results suggest that the [4Fe–4S] cluster could contribute to the formation of a more rigid binding cavity for the diiron subsite.

**FIGURE 3:**
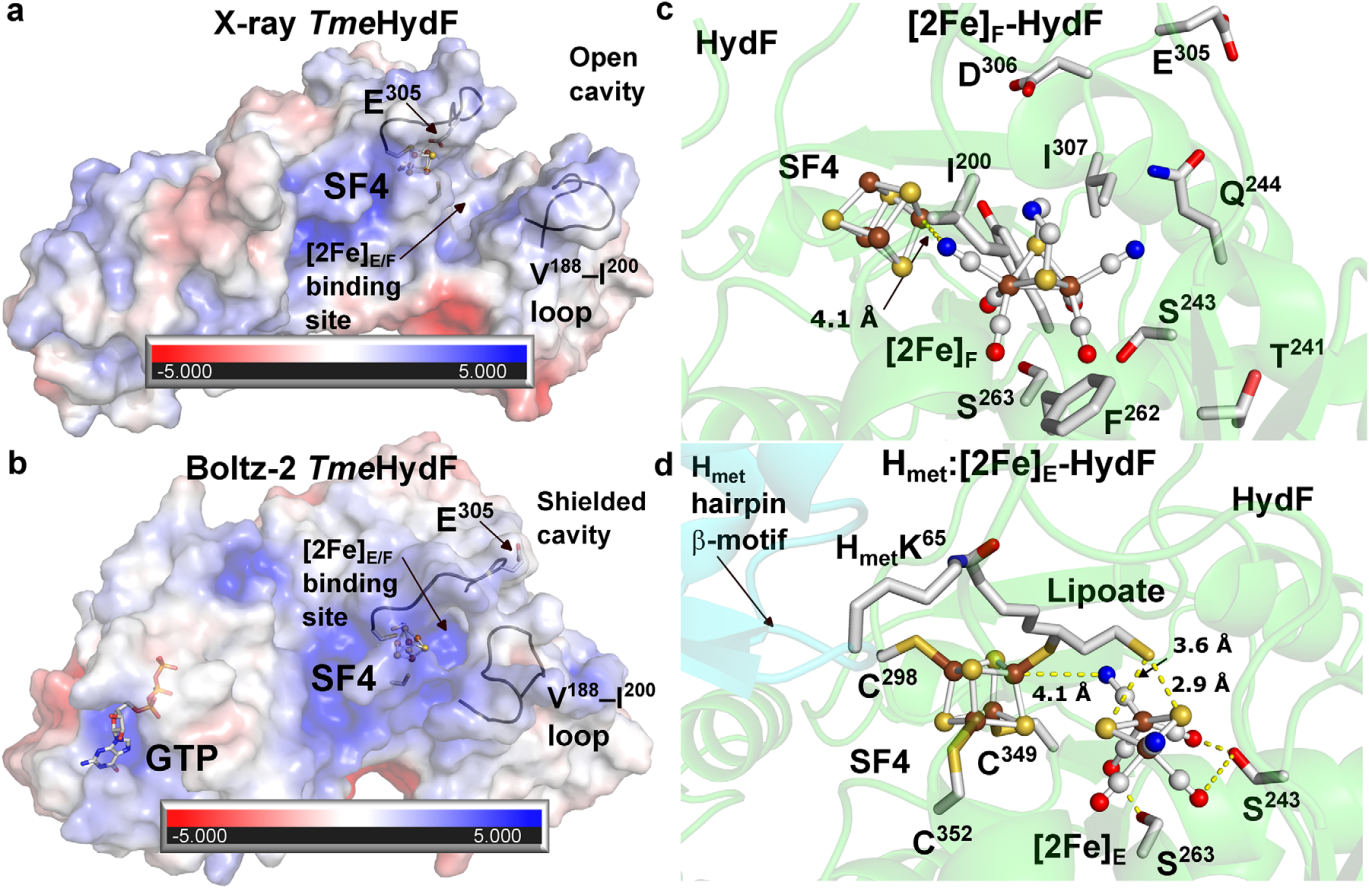
Structural representations of *Tme*HydF and the proposed [2Fe]_E/F_ binding pocket. Electrostatic potential surface representations (±5 kBT/e) of the x‐ray structure of *Tme*HydF (a) and the Boltz‐2 model (b) including the [4Fe–4S] cluster (SF4), GTP (panel b), the flexible loop V^188^–I^200^ (see Figure S7), and the loop carrying E^305^ (cartoon representation). The positively charged binding site of the [2Fe] precursor is shown in blue. (c) Close‐up view of the proposed [2Fe]_F_ binding site. The [4Fe–4S] cluster coordination sphere and residues in proximity to the diiron subsite are shown as sticks (C, grey; N, blue; S, yellow; O, red). (d) Close‐up view of the proposed H_met_:[2Fe]_E_‐HydF complex, highlighting coordination of the lipoate group to the [4Fe–4S] cluster.

While recent docking studies provided valuable initial insight into [2Fe]_E/F_ binding in HydF [31, 34], these models were based on x‐ray coordinates of *Tme*HydF lacking the [4Fe–4S] cluster. Given the proximity of the cubane to the proposed [2Fe] binding cavity, we sought a more realistic representation of the [4Fe–4S]:[2Fe]_F_ interaction. To this end, we employed the recently released Boltz‐2 machine‐learning model [47], which achieves AlphaFold3‐level accuracy in protein–ligand interactions [48], including systems containing complex metal cofactors such as [Fe–S] clusters. A structural model of *Tme*HydF containing bound GTP and a [4Fe–4S] cluster is shown in Figure S6. The model reveals a well‐defined, positively charged pocket suitable for [2Fe]_E/F_ binding, closely matching the binding site inferred from the crystallographic coordinates of *Tme*HydF (Figure 3ab) [26, 31, 34]. Notably, whereas this cavity appears solvent‐exposed in the x‐ray structure, the loop spanning residues V^188^–I^200^ adopts an alternative conformation in the Boltz‐2 model that partially shields the pocket (Figure 3b). Conformational variability of this loop, which may facilitate opening and closing of the binding cavity, was further explored using ColabFold‐AF2 (methods in Supporting Information) and can be visualized in Figure S7 [49, 50]. On this basis, the Boltz‐2 model was used for blind docking simulations with CB‐Dock2 [51], which has recently proven effective in identifying binding sites of maturation intermediates in [NiFe]‐hydrogenases [52]. The most favorable docking site (Vina score –6.8) places the [2Fe]_F_ site in proximity of the [4Fe–4S] cluster near residues Ile^200^, Thr^241^, Glu^242^, Ser^243^, Gln^244^, Phe^262^, Ser^263^, Asp^306^, Ile^307^, and Tyr^376^ (Figure 3c), some of which were recently identified by site‐directed mutagenesis as critical for [2Fe]_E/F_ binding and biosynthesis of the DTMA bridge [34]. These residues form a network of electrostatic, van der Waals/hydrophobic and hydrogen‐bonding interactions stabilizing the diiron subsite within the HydF scaffold. Notably, Glu^305^—which coordinates the [4Fe–4S] cluster in FeS‐*Tme*HydF (Figure 3a)—is displaced away from the cubane in the holo‐HydF model (Figs. 3c), consistent with conformational changes in the binding pocket possibly inferred from NRVS‐detected perturbations of Fe–S vibrational modes upon [2Fe]_F_ binding (Figure 2a). This rearrangement provides a structural rationale for previous mutagenesis studies in which replacement of the conserved Glu^305^(/Asp) showed negligible effects on diiron core binding and assembly [30, 31, 53], explaining why the residue is dispensable for [2Fe]_F_ incorporation despite its conserved position in the cubane‐ligating motif. Importantly, one CN ligand of [2Fe]_F_ is positioned approximately 4 Å from the [4Fe–4S] cluster (Figure 3c), consistent with a non‐covalent, through‐space interaction.

This spatial arrangement is in full agreement with our NRVS data, which exclude Fe–CN vibrational signatures expected for cyanide bridging while revealing subtle perturbations of low‐frequency [4Fe–4S] modes. Next, we combined this structural framework with the recent identification of H_met_ as an essential component of H‐cluster biosynthesis and performed additional structure predictions of HydF (Figure S8) in the presence of GTP, its [4Fe–4S] cluster, the [2Fe]_E_ precursor, and lipoate‐functionalized H_met_ [54], proposed to carry the CH_2_–NH_3_ moiety of the DTMA bridge [17, 19]. The resulting models identify a plausible assembly scenario (Figures 3d and S8) comprising a HydF–H_met_ complex, in which one thiolate of lipoate coordinates to the non‐cysteine‐ligated iron of the cubane, while the second thiolate remains positioned in proximity to the [2Fe]_E_ precursor, consistent with a role in formation of the bridging moiety.

## 3 Conclusion

**FIGURE 4:**
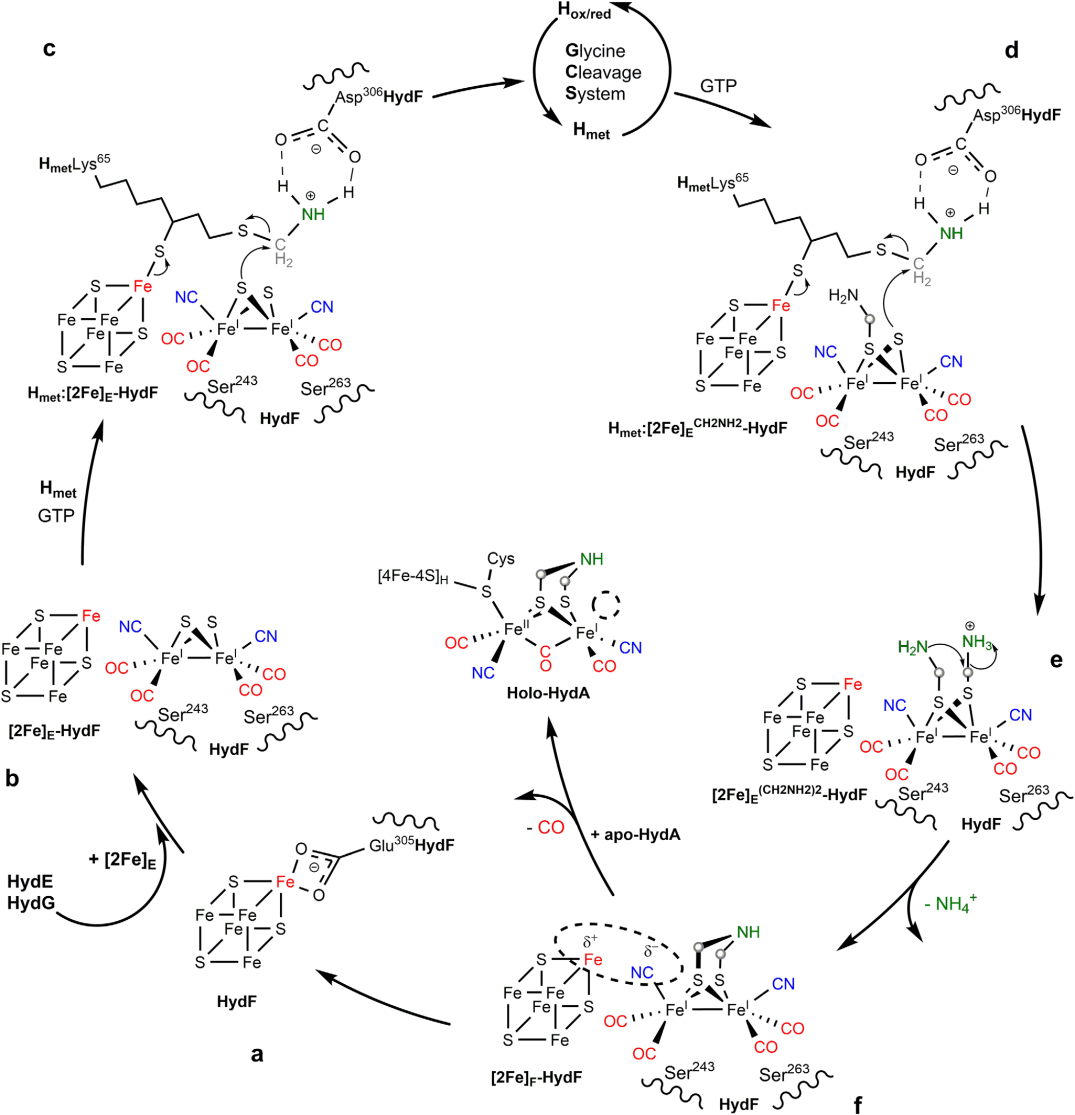
Proposed catalytic cycle of HydF leading to assembly of the fully functional [2Fe]_F_ precursor. (a) [4Fe–4S] cluster of HydF coordinated by a labile glutamate [26]. (b) Transfer of the [2Fe]_E_ precursor by HydE to the diiron‐binding cavity of HydF with concomitant release of the Glu^305^ [4Fe–4S] ligand. (c) Proposed HydF–H_met_ complex in which one thiolate of lipoate coordinates to the [4Fe–4S] cluster, while the second thiol positions the first CH_2_–NH_3_ moiety in proximity to [2Fe]_E_. (d) HydF–H_met_ complex positioning the second CH_2_–NH_3_ moiety in proximity to the [2Fe]_E_^CH2NH2^ intermediate. (e) Intramolecular condensation of the two aminomethyl groups of the [2Fe]_E_^(CH2NH2)2^ intermediate [55]. (f) Holo‐HydF complex showing a noncovalent, through‐space interaction between the [2Fe]_F_ subsite and the cubane. At this stage [2Fe]_F_ is transferred to apo‐HydA, where one CO ligand is released giving rise to the characteristic geometry of the matured H‐cluster. Critical residues Ser^243^, Ser^263^, Glu^305^, and Asp^306^ of *Tme*HydF at different stages of the cycle are highlighted.

Based on these predictions, we outline a tentative mechanistic scenario (Figure 4) that integrates the x‐ray structure of FeS‐HydF (panel **a**) [26], prior work on the role of H_met_ (panels **b**–**d**) [18, 19], and site‐directed mutagenesis studies highlighting the importance of strictly conserved HydF residues Ser^243^ and Ser^263^ for [2Fe]_E/F_ binding and Asp^306^ for biosynthesis of the CH_2_–NH–CH_2_ bridge [34]. This framework incorporates our revised model of the [2Fe]_F_:[4Fe–4S] interaction (panel **f**) and proposes plausible intermediates (panels **c** and **d**), in which H_met_ coordinates to the [4Fe–4S] cluster and delivers DTMA components at the [2Fe]_E_ precursor via its lipoate functionality. In this unified model, the [4Fe–4S] cluster does not coordinate the diiron subsite but instead stabilizes and regulates the binding cavity, controlling access of H_met_ to the fragile [2Fe] precursor during the final stages of H‐cluster assembly. While still preliminary, our analysis provides the first structural framework linking HydF function to earlier maturation stages [31, 32], illustrating how auxiliary Fe–S clusters can guide metallocofactor maturation through structural and electronic preorganization. These findings also establish a basis for future studies aimed at identifying transient intermediates in H‐cluster biosynthesis including the proposed lipoate–[4Fe–4S] cluster interaction in HydF.

## Author Contributions

**Giorgio Caserta**: investigation, conceptualization, software, data curation, methodology, validation, formal analysis, resources, project administration, supervision, visualization, writing – original draft, funding acquisition, writing – review and editing. **Princess Cabotaje**: investigation, data curation, formal analysis, writing – review and editing, methodology, validation, visualization. **Armel T. Waffo**: writing – review and editing, formal analysis, data curation, software. **Deepak Prajapat**: formal analysis, software, methodology. **Ilya Sergueev**: software, formal analysis, supervision, methodology. **Stefan Frielingsdorf**: methodology, software, visualization, writing – review and editing, formal analysis, resources, data curation. **Gustav Berggren**: funding acquisition, project administration, writing – review and editing, resources, investigation, formal analysis, supervision, visualization, validation. The article was written through contributions of all authors.

## Conflicts of Interest

The authors declare no conflict of interest

## Supporting information




**Supporting File**: anie72892‐sup‐0001‐SuppMat.docx.

## Data Availability

Experimental NRVS data; the Boltz‐1 model of *Tme*HydF; CB‐Dock2 blind docking results for the [2Fe]_F_ cofactor within the Boltz‐1 *Tme*HydF model; *Tme*HydF AF2 models (flex configuration); and the Boltz‐2 model of the H_met_–*Tme*HydF complex in the presence of GTP, the [4Fe−4S] cluster, and the [2Fe]_E_ precursor are available on Zenodo (10.5281/zenodo.18669547) [56]. All other data supporting the findings of this study are provided within the manuscript and the Supporting Information.
