# Supplementary material for: A Non‐Covalent [4Fe–4S]/[2Fe] Interface in HydF Guides [FeFe]‐Hydrogenase Maturation
Source: Angew Chem Int Ed Engl. 2026 May 29;65(29):e8078898. doi: 10.1002/anie.8078898 (PMC13360658; doi:10.1002/anie.8078898)
Supplement: Supplementary file 1 — Supporting File: anie72892‐sup‐0001‐SuppMat.docx. [file ANIE-65-e8078898-s001.docx]

**Supporting Information**

**A Non-Covalent [4Fe–4S]/[2Fe] Interface in HydF Guides [FeFe]-Hydrogenase Maturation**

Giorgio Caserta,^a^* Princess R. Cabotaje,^b†^ Armel T. Waffo,^a^ Deepak Prajapat,^c^ Ilya Sergueev,^c^ Stefan Frielingsdorf^a^ and Gustav Berggren^d^*

^a^ Institut für Chemie, Sekr. PC14, Technische Universität Berlin, 10623 Berlin, Germany;

^b^ Department of Chemistry─Ångström Laboratory, Molecular Biomimetics, Uppsala University, 751 20 Uppsala, Sweden
^c^ Deutsches Elektronen-Synchrotron, 22607 Hamburg, Germany;

† current address: Department of Cell and Molecular Biology – Molecular Evolution, Uppsala University, 751 24 Uppsala, Sweden; Okinawa Institute of Science and Technology – Evolution, Cell Biology, and Symbiosis Unit, 904-0495 Okinawa, Japan

* E-mail: [giorgio.caserta@tu-berlin.de](mailto:giorgio.caserta@tu-berlin.de), [gustav.berggren@kemi.uu.se](mailto:gustav.berggren@kemi.uu.se)

**Table of Contents**

**Methods**

**Preparation of ^57^Fe solution**

**HydF overproduction and purification**

**Demetallation of HydF and Reconstitution of [4Fe–4S] cluster in apo-HydF**

**Preparation of apo/holo-HydF-[2Fe]_F_**

**Protein and Fe content**

**UV-visible spectroscopy**

**IR spectroscopy**

**Nuclear resonance vibrational spectroscopy**

**3D Structure Predictions**

**CB-Dock2 Cavity Detection-Guided Blind Docking**

**Supplementary figures**

**Figure S1.** **UV-Visible absorption spectrum of ^57^FeS-*Tm*HydF.**

**Figure S2. IR spectroscopic characterization of holo-*Tm*HydF.**

**Figure S3. UV-Visible absorption spectra of [4^57^Fe–4S]-[2Fe]_F_ and [4^56^Fe–4S]-[2^57^Fe]_F_ holo-*Tm*HydF.**

**Figure S4. NRVS characterization of as isolated and reduced ^57^FeS-HydF.**

**Figure S5. UV-Visible absorption spectra apo-[2^57^Fe]_F_-*Tm*HydF and apo-*Tm*HydF.**

**Figure S6. Boltz-2 model of *Tme*HydF.**

**Figure S7.** **Conformational variability in *Tme*HydF structural models.**

**Figure S8.** **Boltz-2 structural prediction of the HydF–H_met_ complex**.

**Supplementary references**

**Preparation of ^57^Fe solution**

^57^Fe metal powder (50 mg, ~95% isotopic enrichment) was dissolved overnight under aerobic conditions in 1.4 mL of 20% (v/v) H_2_SO_4_ (prepared by mixing 0.3 mL concentrated H_2_SO_4_ with 1.1 mL H_2_O). This procedure yielded a 0.62 M Fe^2+^ stock solution, which was subsequently basified with 1.0 M Tris buffer (pH 8.0) and used directly for iron–sulfur cluster reconstitution assays at a final Fe^2+^ concentration of 0.023 M.

**HydF overproduction and purification**

Isolation of HydF from *T. maritima* was performed as previously described with minor modifications.^[1,2]^ Initially, small scale overproduction tests were performed on Rosetta (I) and Rosetta (II) competent cells transformed with a *T. maritima* HydF-encoding expression vector, with the positive clones selected based on kanamycin resistance.^[2]^ Both constructs exhibited comparable overproduction levels of soluble protein and Rosetta (I) was selected for subsequent large‑scale production of HydF due to its consistently faster growth rate, as determined by optical density (OD_600_) measurements

Precultures were grown overnight in 10 mL LB medium containing 100 μg/mL ampicillin and 50 μg/mL kanamycin at 37°C. These cultures were subsequently used to inoculate Miller’s modified LB medium containing 100 μg/mL ampicillin and 50 μg/mL kanamycin. Cultures were grown at 37 °C and 150 rpm until an OD_600_ of approximately 0.4–0.6 was reached. HydF production was induced with 0.5 mM IPTG (isopropyl β-d-1-thiogalactopyranoside). After 5 hours, the cells were harvested by centrifugation (4500 rpm, 4 °C, 10 min). The cell pellet was resuspended in a solution containing 150 mM NaCl and 50 mM Tris–HCl, pH 8.0, and centrifuged again (5000 rpm, 20 min, 4 °C) to remove the residual media. The cell paste was frozen in liquid nitrogen and kept at −80 °C until further use.

For purification, the cells were thawed in the presence of lysozyme from chicken egg white (0.6 mg/mL), DNAse I from bovine pancreas (0.05 mg/mL), RNase A from bovine pancreas (0.05 mg/mL) and 0.5 % Triton X-100, and discontinuously sonicated until the solution became clear (15 min). The solution was ultracentrifuged (55,000 rpm, 90 min, 4 °C). The supernatant from the ultracentrifugation step was separated into 30-ml aliquots in falcon tubes, which were heated to 75 °C for 12 min to denature the *E. coli* host proteins. The precipitated proteins were separated by centrifugation (15,000 rpm, 15 min, 4 °C), and the pellet was discarded. The supernatant was used for ammonium sulfate precipitation, where crystalline ammonium sulfate was added in small portions to the continuously stirred solution at 4 °C. After the final addition of ammonium sulfate (65% saturation concentration), the cloudy solution was gently stirred at 4 °C for 45 min before centrifugation (15,000 rpm, 4 °C, 15 min). The resulting off-white pellet was resuspended in Buffer A solution containing 1 M ammonium sulfate, 150 mM NaCl, and 50 mM Tris–HCl, pH 8.0 under stirring. The filtrated protein solution was loaded onto HiPrep^TM^ Phenyl HP 16/10 column (Cytiva) column equilibrated with Buffer A, and the flowthrough was collected. The column was washed with Buffer A, removing all nucleic acids and remaining *E. coli* contaminants, until the absorbance at 280 nm reached the baseline. *Tm*HydF was eluted with a gradient from 100 % Buffer A to 100 % Buffer B (150 mM NaCl, 50 mM Tris–HCl, pH 8.0). The protein content in the elution fractions was verified using SDS-PAGE gel, and the HydF-containing fractions were collected and concentrated using 30-kDa Centricon filters (Amicon). The purified protein was flash-frozen in liquid nitrogen and stored at −80 °C.

**Demetallation of HydF and reconstitution of [4Fe–4S] cluster in apo-HydF**

Demetallation of HydF from *T. maritima* was performed as previously described with minor modifications.^[1,2]^ The apo form of *Tm*HydF was generated by overnight treatment of the protein with 10 mM EDTA under reducing conditions (2 mM sodium dithionite) at 4 °C in order to remove any residual metal ions. Apo-protein was isolated by running the reaction mixture through a PD-10 column (GE Healthcare), equilibrated with Buffer B (150 mM NaCl, 100 mM Tris-HCl pH 8.0). The [Fe–S] cluster reconstitution of apo-HydF was conducted in anaerobic conditions inside a glove box (O_2_ < 0.5 ppm) under nitrogen atmosphere. The apo-protein, normally 50 µM, was incubated in Buffer B (150 mM NaCl, 50 mM Tris–HCl, pH 8.0), with 10 mM DTT for 10 min at 20 °C, followed by 5/5.5 molar excess Mohr's salt or ferrous ammonium sulfate [(NH_4_)_2_Fe(SO_4_)_2_·6H_2_O] (or ^57^FeSO_4_, see above) and L-cysteine. The reaction is initiated by the addition of a catalytic amount of the *E. coli* cysteine desulfurase CsdA (1 % molar equivalent). All the reactions were normally conducted overnight. The reconstituted proteins were then centrifuged 30 min at 12000 rpm and purified using size exclusion chromatography via Superdex™ 200 10/300 GL (Cytiva) equilibrated with Buffer B, 150 mM NaCl, 50 mM Tris–HCl, pH 8.0 (supplemented with 5 mM DTT). Elution fractions containing the FeS-*Tm*HydF protein were pooled and concentrated using Amicon 30-kDa centrifugal filters. The protein was finally aliquoted, flash-frozen, and stored at −80 °C.

**Preparation of apo/holo-HydF-[2Fe]_F_**

[2Fe]_F_ was synthesized following published procedures.^[3,4]^ ^56/57^FeS-HydF and apo-hydF were reacted with 8–10 molar excess of [2^56/57^Fe]_F_ (after removal of DTT via buffer exchange done 4x) for 1.0 h in Buffer B as described above. Excess chemicals were removed with a desalting column (PD-10 column, GE Healthcare). The proteins were then concentrated to ca 1.5 mM and stored in liquid N_2_ till beamtime measurements. Incorporation of the complexes into the FeS–HydF protein was ascertained via UV-visible absorption spectroscopy (**Fig. S3**), Fe quantitation and FTIR spectroscopy (**Fig. S2**).

**Protein and Fe content**

Protein estimations were performed via Bradford assay using bovine serum albumin as a standard.^[5]^ Fe contents for apo-HydF treated with [2^57^Fe]_F_ and ^57^FeS-HydF were determined according to the methods of Fish.^[6]^

**UV-visible spectroscopy**

UV-Vis spectra of FeS-HydF, holo/apo-HydF, apo-HydF treated with [2^57^Fe]_F_ were obtained using an AvaSpec-ULS2048-USB2-UA-50: Avantes Fiber Optic UV/VIS/NIR spectrometer connected to the cell holder located in a glovebox with optical fibers.

**IR spectroscopy**

For IR measurements, 8-10 µl of holo-HydF ([2^57^Fe]-FeS-HydF) in 50 mM Tris-HCl, 150 mM NaCl, pH 8.0 (at 10 °C) were transferred into a homemade, gas-tight and temperature-controlled (10 °C) transmission cell equipped with two sandwiched CaF_2_ windows that are separated by a Teflon spacer (50 µm path length). Spectra were recorded by averaging 200 scans using a Tensor 27 Fourier-transform spectrometer (Bruker) equipped with a liquid nitrogen-cooled mercury-cadmium-telluride detector. The Bruker OPUS software 7.8 was used to acquire and analyze the data. A buffer spectrum was used as reference to calculate the corresponding absorbance spectra. OriginPro 2021 software was used to prepare figures.

**Nuclear resonance vibrational spectroscopy (NRVS)**

Freshly reconstituted ^57^FeS-HydF, dithionite-treated ^57^FeS-HydF, [2Fe]-^57^FeS-HydF and [2^57^Fe]-FeS-HydF and [2^57^Fe]-apo-HydF samples were concentrated to ca 1.2-1.5 mM and a volume of about 30 µl was filled into a Kapton tape-sealed copper cell, flash frozen and stored in liquid nitrogen until the start of beamtime measurements. NRVS measurements were conducted at Petra III P01 (Germany, [http://petra3.desy.de](http://petra3.desy.de/)) according to preceding investigations. Raw NRVS data were converted to single-phonon ^57^Fe partial vibrational densities of states (PVDOS) using the PHOENIX software package (<https://www.spectra.tools/>).^[7]^ The energy scales were calibrated with a [NEt_4_][^57^FeCl_4_] sample. The temperature of the samples was maintained at ca 12 K using a liquid He cryostat. To emphasize the region of interest, sectional measurements of the spectral regions were performed. Each scan was divided into two segments with different data collection times (second per point, s/pt). We used 5 s/pt for the region from –80 to 320 cm^–1^ and 9 s/pt for the region from 320 to 720 cm^–1^. Experimental data are available on Zenodo at [10.5281/zenodo.18669547](https://doi.org/10.5281/zenodo.18669547).

**3D Structure Predictions**

Boltz-1/ Boltz-2 were used to generate a structural model of *Thermosipho melanesiensis* HydF containing a bound [4Fe–4S] cluster and GTP (<https://colab.research.google.com/github/sokrypton/ColabFold/blob/main/Boltz1.ipynb>).^[8]^ For predictions of the HydF-H_met_ complex including the lipoate, a Boltz-2 instance (version 2.2.1) locally installed on the high performance cluster of TU Berlin was employed.^[9]^ This machine-learning approach proved more suitable than the recently released AlphaFold3,^[10]^ as it allows explicit incorporation of complex metal cofactors into the structural model. For prediction of conformational variability, we used ColabFold-AF2 (v1.5.5)^[11,12]^ and the following settings were applied: “max_msa” was set to “32:64”, “num_seeds” was set to “16”, and “use_dropouts” was not activated (HydF_flex is available on Zenodo at [10.5281/zenodo.18669547](https://doi.org/10.5281/zenodo.18669547)).^[13]^ All predictions employed automatic MSA generation.^[11]^ The HydF protein sequence from *Thermosipho melanesiensis* was retrieved from ref.^[14]^ Structural analysis and figure generation were performed using PyMOL.

**CB-Dock2 Cavity Detection-Guided Blind Docking**

Blind docking simulations were performed using the CB-Dock2 online platform.^[15,16]^ Boltz-1/2–derived coordinates of *T. melanesiensis* HydF were used as receptor structures, while the coordinates of the [2Fe]_F_ cofactor served as the ligand. CB-Dock2 identifies potential binding cavities across the entire protein and performs docking within each cavity using AutoDock Vina. The resulting receptor–ligand complex is provided as PDB file (available on Zenodo at [10.5281/zenodo.18669547](https://doi.org/10.5281/zenodo.18669547)).


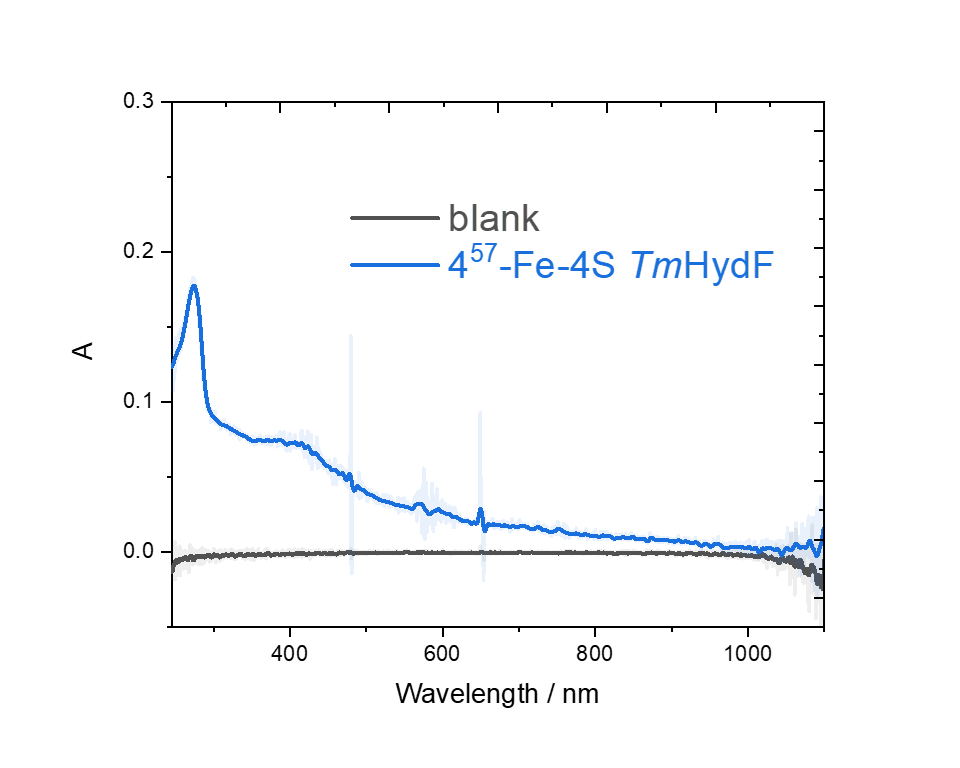


**Figure S1.** UV-Visible absorption spectra of Buffer B (150 mM NaCl, 100 mM Tris-HCl pH 8.0) (“blank”, grey trace) and [4^57^Fe–4S] *Tm*HydF (*ca.* 50 µM) in blue trace.


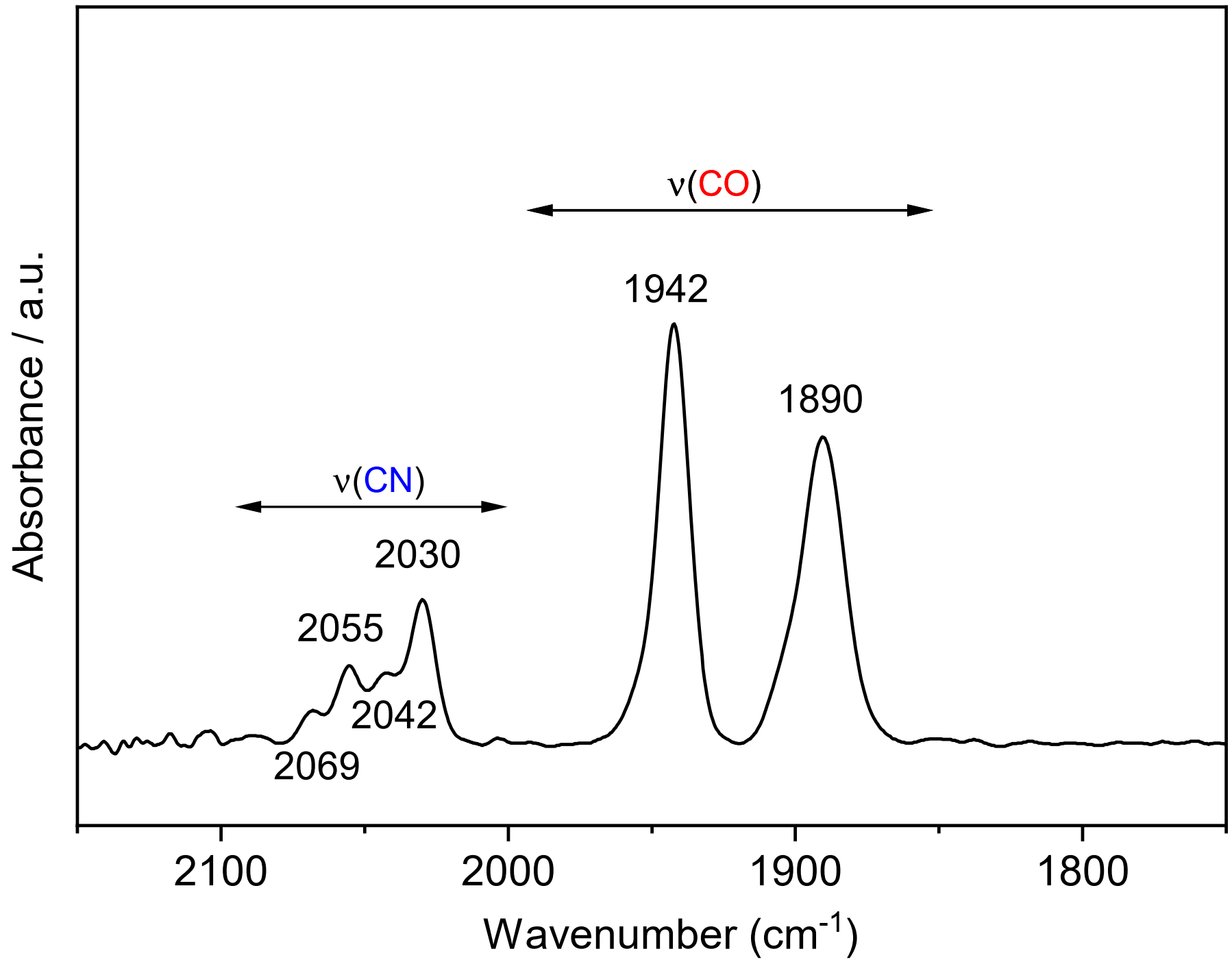


**Figure S2**. **IR spectroscopic characterization of holo-*Tm*HydF**. IR spectrum of holo-*Tm*HydF ([2Fe]_F_-^57^FeS-HydF) with absorptions related to the stretching vibrations of the CO and CN^−^ ligands of the [2Fe]_F_ site. Peak positions are labeled with the corresponding wavenumbers.


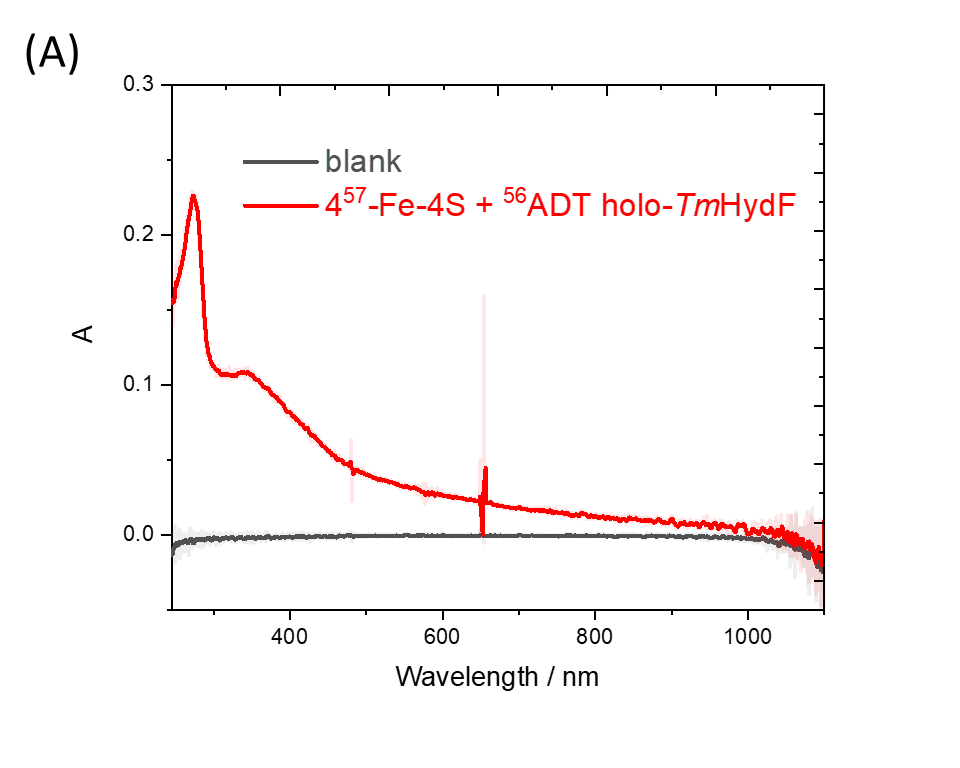


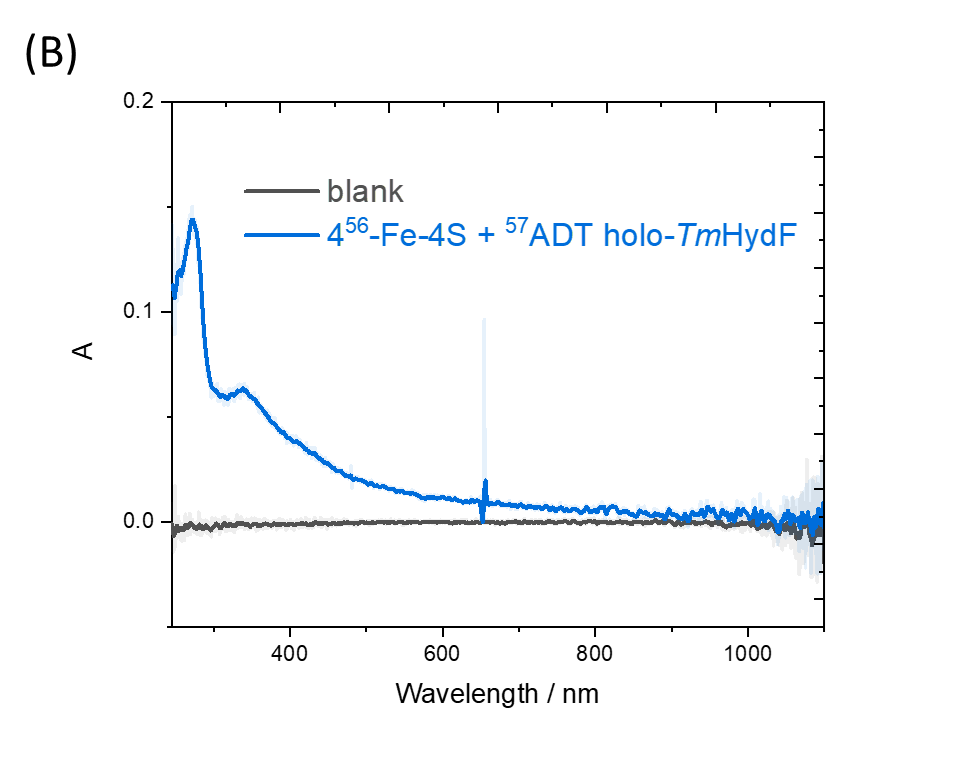


**Figure S3.** UV-Visible absorption spectra of Buffer B (150 mM NaCl, 100 mM Tris-HCl pH 8.0) (“blank”, grey trace); (A) [4^57^Fe–4S] + ^56^ADT holo-*Tm*HydF (50 µM) in red trace and (B) [4^56^Fe–4S] + ^57^ADT holo-*Tm*HydF (30 µM) in blue trace.


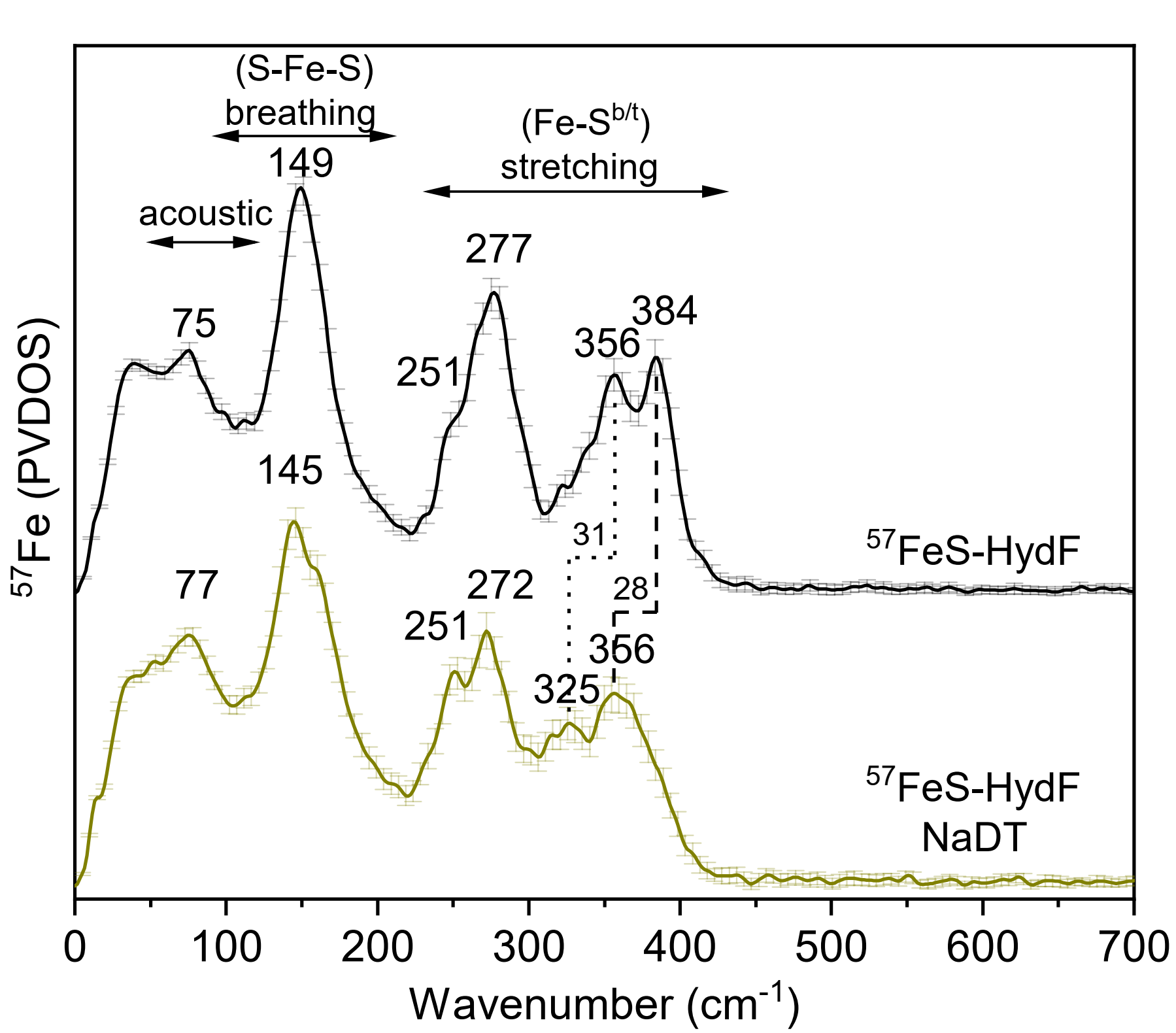


**Figure S4.** **NRVS characterization of as isolated and reduced ^57^FeS-HydF.**FeS-HydF exhibits typical absorptions of a [4Fe4S]-cluster characterized by acoustic (<100 cm^–1^), breathing (bending and twisting, 100–200 cm^–1^) and Fe-S stretching (250–400 cm^–1^) of terminal (S^t^) and bridging (S^b^) sulfides. These data resemble preceding data collected on the [4Fe–4S] cluster of the ferredoxin from *Pyrococcus furiosus*,^[17]^ the nitrogenase Fe protein from *Azotobacter vinelandii*,^[18]^ the IspH protein from *E. coli*,^[19]^ and NO-sensing proteins WhiD and NsrR from *S. coelicolor* ^[20]^ as well as synthetic [4Fe–4S] analogs suggesting a minor influence of protein modes on the cluster vibrations.^[21,22]^


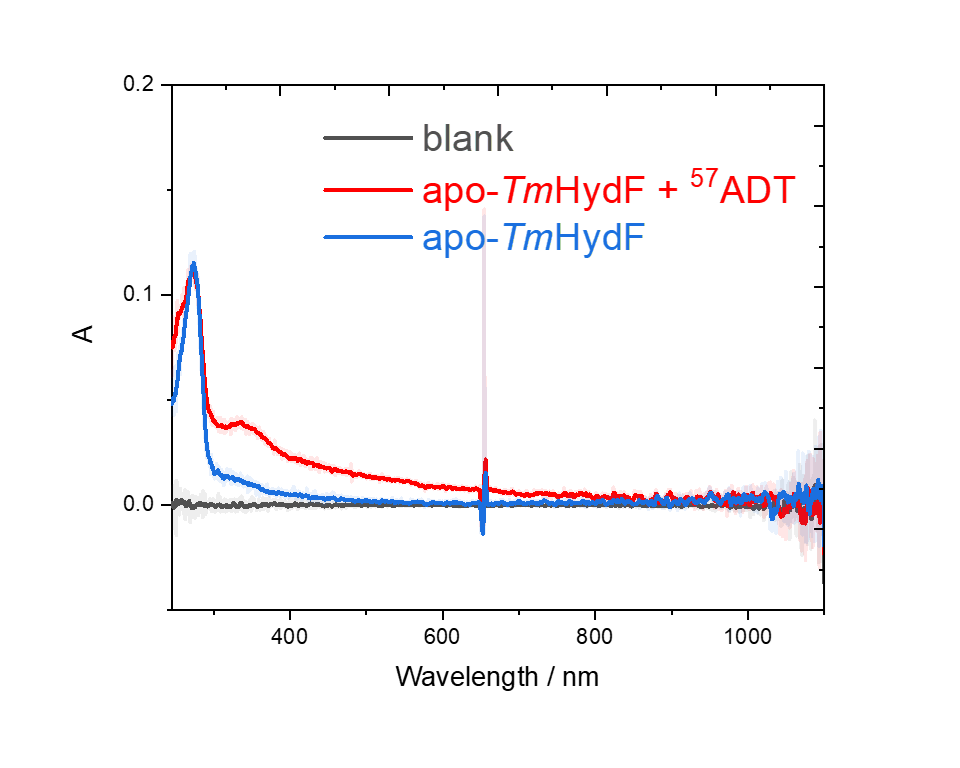


**Figure S5.** UV-Visible absorption spectra of Buffer B (150 mM NaCl, 100 mM Tris-HCl pH 8.0) (“blank”, grey trace), apo-*Tm*HydF (25 µM) + ^57^ADT in red trace and (B) apo-*Tm*HydF (25 µM) in blue trace.


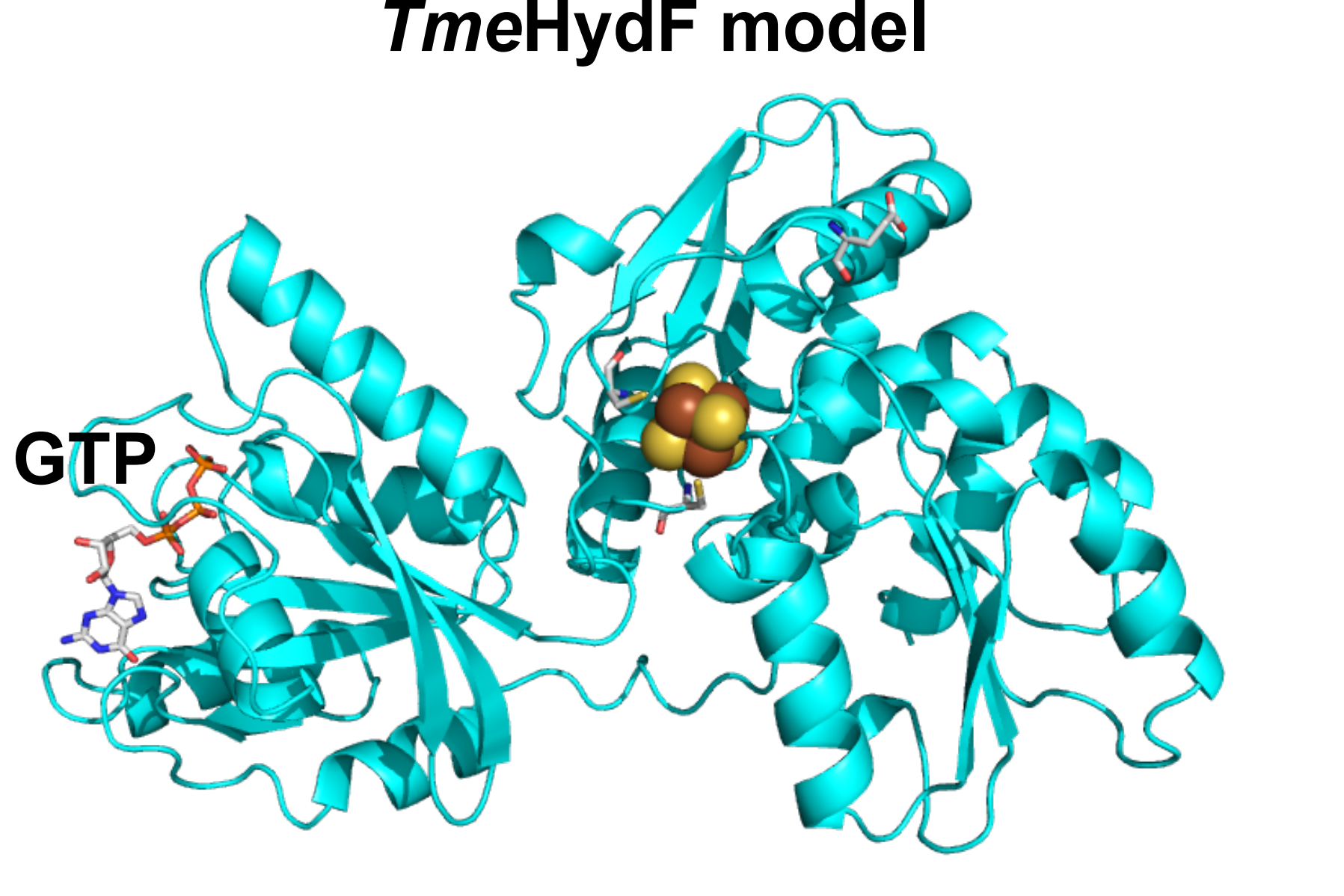


**Figure S6. Boltz-2 model of *Tme*HydF.** Cartoon representation of the predicted *Tme*HydF model including bound GTP (sticks; C, grey; N, blue; O, red; P, orange) and the [4Fe–4S] cluster (spheres; Fe, brown; S, yellow) ligated by the three conserved cysteine residues.


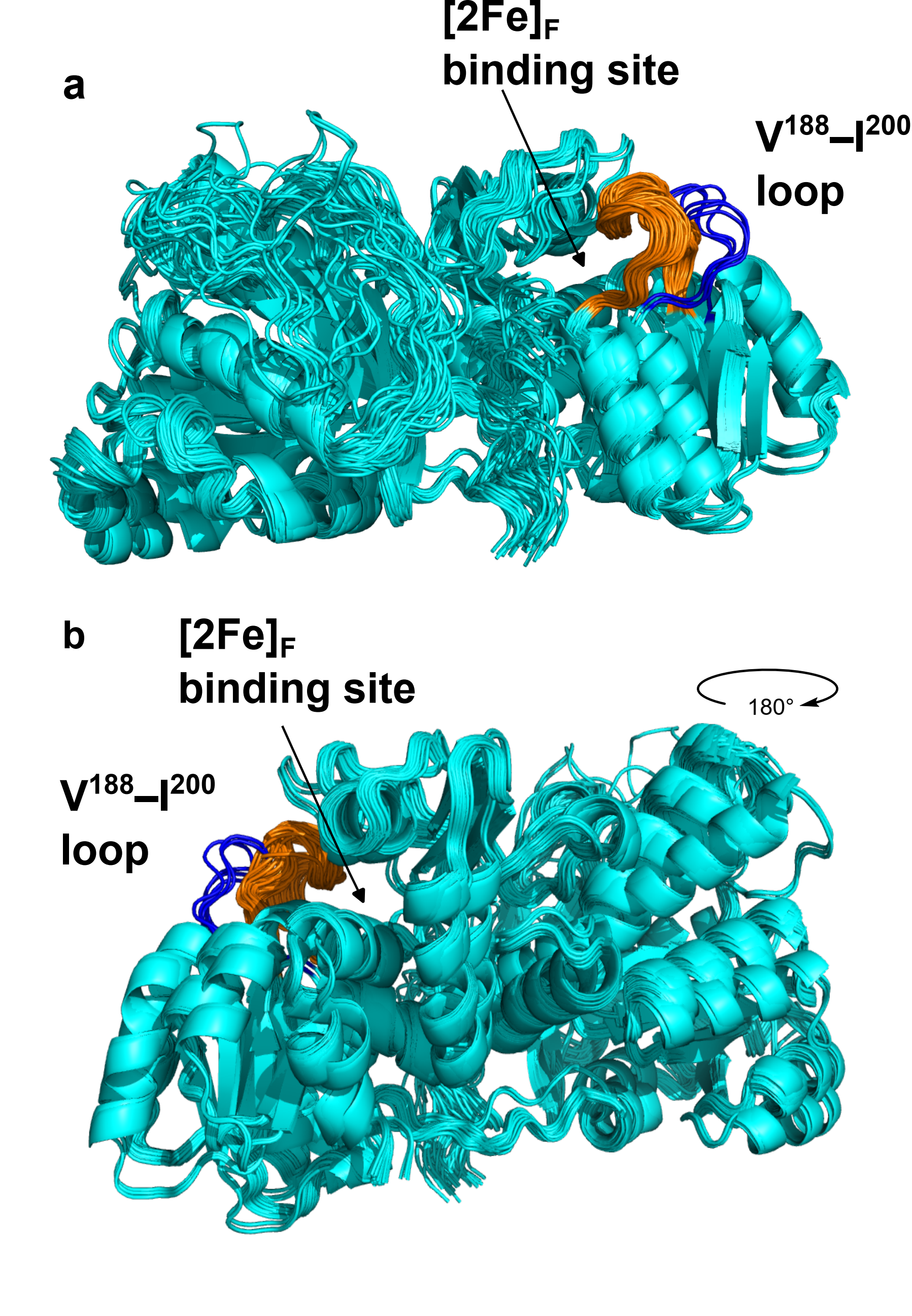


**Figure S7**. **Conformational variability in *Tme*HydF structural models.** Front (**a**) and back (**b**) views of an overlay of structural models of *Tme*HydF predicted by AlphaFold2 using different presets (see Methods). The loop spanning residues V^188^–I^200^ exhibits pronounced conformational flexibility and adopts distinct conformations. In a closed state (orange cartoon), the loop shields the proposed [2Fe]_F_ binding cavity (see also **Fig. 3b**), whereas in an open state (blue cartoon) the pocket is solvent exposed, closely resembling the conformation observed in the X-ray structure (**Fig. 3a**). While the overall HydF fold is conserved across all models, regions comprising residues 1–10, 36–48, and 66–80 (left side of the representations in panel **a**) display increased flexibility, consistent with their absence or poor definition in the crystallographic structure (PDB: 5KH0).^[14]^


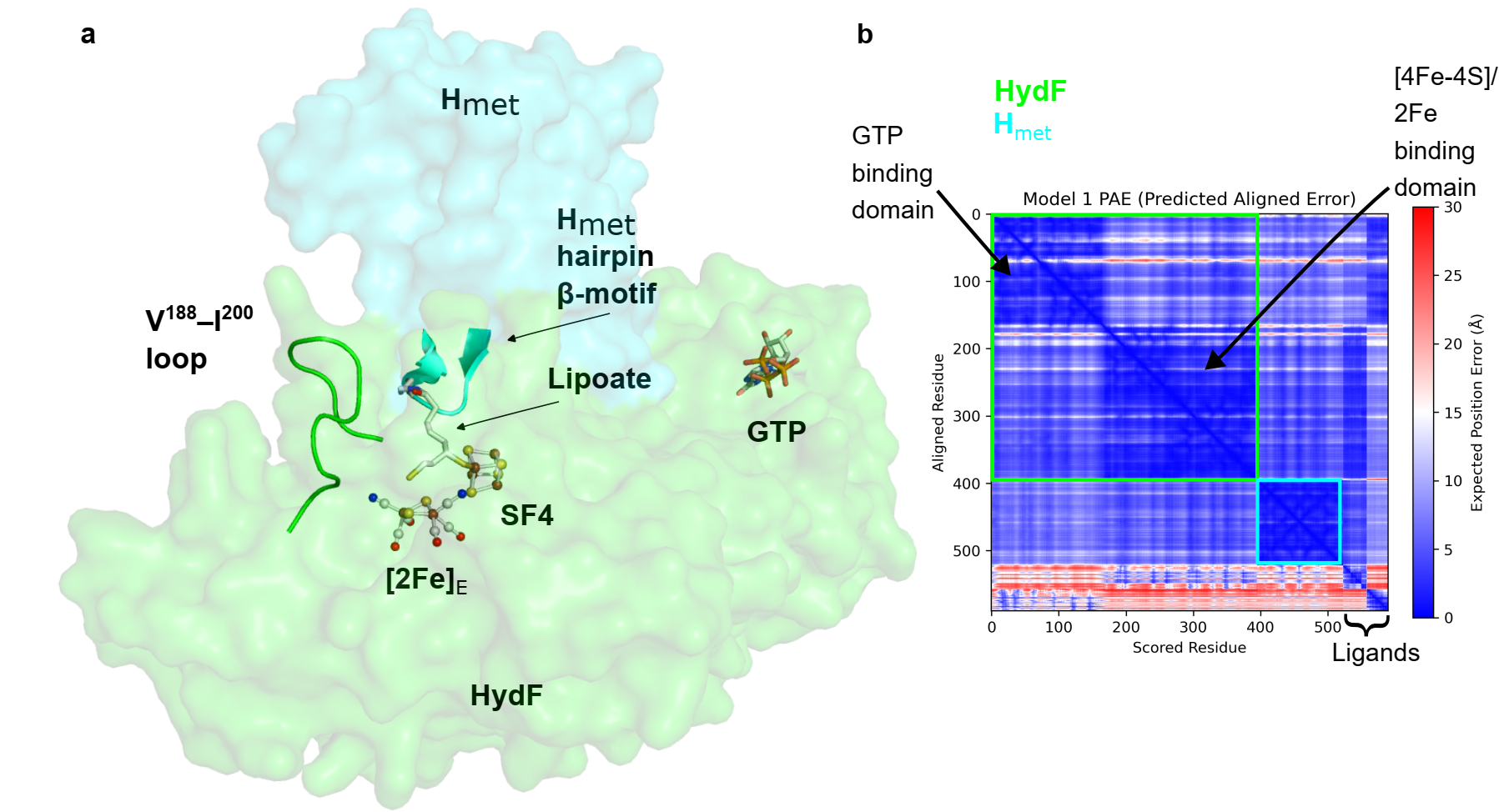


**Figure S8**. (**a**) Boltz-2 model of *Tme*HydF (green surface) in complex with GTP (stick representation), the [4Fe–4S] cluster and [2Fe]_E_ precursor (both shown as sticks and spheres), and lipoate-functionalized H_met_ (cyan surface). Lys^65^, located within a β-hairpin motif, carries the lipoate moiety via an amide linkage between its ε-NH_2_ group and the carboxylate of lipoic acid (shown as sticks). (**b**) Predicted aligned error (PAE) analysis of the H_met_:[2Fe]_E_–HydF complex shown in (**a**). The PAE reports the confidence of the Boltz-2 model in the relative positioning of residue pairs within the complex. Values are given in Å, ranging from 0 (blue, high confidence) to 30 (red, low confidence), and are displayed as a heat map. Areas of HydF and H_met_ are highlighted by squares (color code as in **a**).

**Supplementary references**

[1] G. Berggren, R. Garcia-Serres, X. Brazzolotto, M. Clemancey, S. Gambarelli, M. Atta, J.-M. Latour, H. L. Hernández, S. Subramanian, M. K. Johnson, M. Fontecave, “An EPR/HYSCORE, Mössbauer, and resonance Raman study of the hydrogenase maturation enzyme HydF: a model for N-coordination to [4Fe–4S] clusters” *J Biol Inorg Chem* **2014**, *19*, 75–84.

[2] B. Németh, H. Land, A. Magnuson, A. Hofer, G. Berggren, “The maturase HydF enables [FeFe] hydrogenase assembly via transient, cofactor-dependent interactions” *Journal of Biological Chemistry* **2020**, *295*, 11891–11901.

[3] H. Li, T. B. Rauchfuss, “Iron Carbonyl Sulfides, Formaldehyde, and Amines Condense To Give the Proposed Azadithiolate Cofactor of the Fe-Only Hydrogenases” *J. Am. Chem. Soc.* **2002**, *124*, 726–727.

[4] R. Gilbert-Wilson, J. F. Siebel, A. Adamska-Venkatesh, C. C. Pham, E. Reijerse, H. Wang, S. P. Cramer, W. Lubitz, T. B. Rauchfuss, “Spectroscopic Investigations of [FeFe] Hydrogenase Maturated with [^57^ Fe_2_ (adt)(CN)_2_ (CO)_4_ ]^2–^” *J. Am. Chem. Soc.* **2015**, *137*, 8998–9005.

[5] M. M. Bradford, “A rapid and sensitive method for the quantitation of microgram quantities of protein utilizing the principle of protein-dye binding” *Analytical Biochemistry* **1976**, *72*, 248–254.

[6] W. W. Fish in *Methods in Enzymology*, Elsevier, **1988**, pp. 357–364.

[7] L. B. Gee, H. Wang, S. P. Cramer in *Methods in Enzymology*, Elsevier, **2018**, pp. 409–425.

[8] J. Wohlwend, G. Corso, S. Passaro, N. Getz, M. Reveiz, K. Leidal, W. Swiderski, L. Atkinson, T. Portnoi, I. Chinn, J. Silterra, T. Jaakkola, R. Barzilay, **2024**, Biophysics preprint, DOI: 10.1101/2024.11.19.624167.

[9] S. Passaro, G. Corso, J. Wohlwend, M. Reveiz, S. Thaler, V. R. Somnath, N. Getz, T. Portnoi, J. Roy, H. Stark, D. Kwabi-Addo, D. Beaini, T. Jaakkola, R. Barzilay, **2025**, Molecular Biology preprint, DOI: 10.1101/2025.06.14.659707.

[10] J. Abramson, J. Adler, J. Dunger, R. Evans, T. Green, A. Pritzel, O. Ronneberger, L. Willmore, A. J. Ballard, J. Bambrick, S. W. Bodenstein, D. A. Evans, C.-C. Hung, M. O’Neill, D. Reiman, K. Tunyasuvunakool, Z. Wu, A. Žemgulytė, E. Arvaniti, C. Beattie, O. Bertolli, A. Bridgland, A. Cherepanov, M. Congreve, A. I. Cowen-Rivers, A. Cowie, M. Figurnov, F. B. Fuchs, H. Gladman, R. Jain, Y. A. Khan, C. M. R. Low, K. Perlin, A. Potapenko, P. Savy, S. Singh, A. Stecula, A. Thillaisundaram, C. Tong, S. Yakneen, E. D. Zhong, M. Zielinski, A. Žídek, V. Bapst, P. Kohli, M. Jaderberg, D. Hassabis, J. M. Jumper, “Accurate structure prediction of biomolecular interactions with AlphaFold 3” *Nature* **2024**, *630*, 493–500.

[11] M. Mirdita, K. Schütze, Y. Moriwaki, L. Heo, S. Ovchinnikov, M. Steinegger, “ColabFold: making protein folding accessible to all” *Nat Methods* **2022**, *19*, 679–682.

[12] J. Jumper, R. Evans, A. Pritzel, T. Green, M. Figurnov, O. Ronneberger, K. Tunyasuvunakool, R. Bates, A. Žídek, A. Potapenko, A. Bridgland, C. Meyer, S. A. A. Kohl, A. J. Ballard, A. Cowie, B. Romera-Paredes, S. Nikolov, R. Jain, J. Adler, T. Back, S. Petersen, D. Reiman, E. Clancy, M. Zielinski, M. Steinegger, M. Pacholska, T. Berghammer, S. Bodenstein, D. Silver, O. Vinyals, A. W. Senior, K. Kavukcuoglu, P. Kohli, D. Hassabis, “Highly accurate protein structure prediction with AlphaFold” *Nature* **2021**, *596*, 583–589.

[13] G. Kim, S. Lee, E. L. Karin, H. Kim, Y. Moriwaki, S. Ovchinnikov, M. Steinegger, M. Mirdita, "Easy and Accurate Protein Structure Prediction Using ColabFold,” *Nature Protocols* **2025**, *20*, 620–642.

[14] G. Caserta, L. Pecqueur, A. Adamska-Venkatesh, C. Papini, S. Roy, V. Artero, M. Atta, E. Reijerse, W. Lubitz, M. Fontecave, “Structural and functional characterization of the hydrogenase-maturation HydF protein” *Nat Chem Biol* **2017**, *13*, 779–784.

[15] Y. Liu, X. Yang, J. Gan, S. Chen, Z.-X. Xiao, Y. Cao, “CB-Dock2: improved protein–ligand blind docking by integrating cavity detection, docking and homologous template fitting” *Nucleic Acids Research* **2022**, *50*, W159–W164.

[16] X. Yang, Y. Liu, J. Gan, Z.-X. Xiao, Y. Cao, “FitDock: protein–ligand docking by template fitting” *Briefings in Bioinformatics* **2022**, *23*, bbac087.

[17] D. Mitra, V. Pelmenschikov, Y. Guo, D. A. Case, H. Wang, W. Dong, M.-L. Tan, T. Ichiye, F. E. Jenney, M. W. W. Adams, Y. Yoda, J. Zhao, S. P. Cramer, “Dynamics of the [4Fe-4S] Cluster in *Pyrococcus furiosus* D14C Ferredoxin via Nuclear Resonance Vibrational and Resonance Raman Spectroscopies, Force Field Simulations, and Density Functional Theory Calculations” *Biochemistry* **2011**, *50*, 5220–5235.

[18] D. Mitra, S. J. George, Y. Guo, S. Kamali, S. Keable, J. W. Peters, V. Pelmenschikov, D. A. Case, S. P. Cramer, “Characterization of [4Fe-4S] cluster vibrations and structure in nitrogenase Fe protein at three oxidation levels via combined NRVS, EXAFS, and DFT analyses” *J Am Chem Soc* **2013**, *135*, 2530–2543.

[19] I. Faus, A. Reinhard, S. Rackwitz, J. A. Wolny, K. Schlage, H. Wille, A. Chumakov, S. Krasutsky, P. Chaignon, C. D. Poulter, M. Seemann, V. Schünemann, “Isoprenoid Biosynthesis in Pathogenic Bacteria: Nuclear Resonance Vibrational Spectroscopy Provides Insight into the Unusual [4Fe‐4S] Cluster of the *E. coli* LytB/IspH Protein” *Angew Chem Int Ed* **2015**, *54*, 12584–12587.

[20] P. N. Serrano, H. Wang, J. C. Crack, C. Prior, M. I. Hutchings, A. J. Thomson, S. Kamali, Y. Yoda, J. Zhao, M. Y. Hu, E. E. Alp, V. S. Oganesyan, N. E. Le Brun, S. P. Cramer, “Nitrosylation of Nitric‐Oxide‐Sensing Regulatory Proteins Containing [4Fe‐4S] Clusters Gives Rise to Multiple Iron–Nitrosyl Complexes” *Angew Chem Int Ed* **2016**, *55*, 14575–14579.

[21] H. Wang, V. Pelmenschikov, Y. Yoda, S. P. Cramer, “NRVS of Fe S cluster proteins & models – A bestiary of nifty normal modes” *Journal of Inorganic Biochemistry* **2025**, *270*, 112935.

[22] L. Grunwald, V. Pelmenschikov, H. Wang, Y. Yoda, N. Nagasawa, K. Tamasaku, S. P. Cramer, V. Mougel, “Vibrational Architecture of [Fe_4_ S_4_ ]^0/1+/2+/3+/4+^ Iron–Sulfur Cubanes” *Inorg. Chem.* **2025**, *64*, 18062–18067.
